# The critical periods of cerebral plasticity: A key aspect in a dialog between psychoanalysis and neuroscience centered on the psychopathology of schizophrenia

**DOI:** 10.3389/fnmol.2022.1057539

**Published:** 2022-12-14

**Authors:** Jessica Tran The, Pierre J. Magistretti, Francois Ansermet

**Affiliations:** ^1^INSERM U1077 Neuropsychologie et Imagerie de la Mémoire Humaine, Caen, France; ^2^Ecole Pratique des Hautes Etudes, Université Paris Sciences et Lettres, Paris, France; ^3^UFR de Psychologie, Université de Caen Normandie, Caen, France; ^4^Centre Hospitalier Universitaire de Caen, Caen, France; ^5^Cyceron, Caen, France; ^6^Agalma Foundation Geneva, Chemin des Mines, Switzerland; ^7^Brain Mind Institute, Swiss Federal Institute of Technology Lausanne, Lausanne, Switzerland; ^8^Division of Biological and Environmental Sciences and Engineering (BESE), King Abdullah University of Science and Technology, Thuwal, Saudi Arabia; ^9^Département de Psychiatrie, Faculté de Médecine, Université de Genève, Geneva, Switzerland

**Keywords:** schizophrenia, psychoanalysis, cerebral plasticity, environment, psychosis

## Abstract

Through research into the molecular and cellular mechanisms that occur during critical periods, recent experimental neurobiological data have brought to light the importance of early childhood. These have demonstrated that childhood and early environmental stimuli play a part not only in our subjective construction, but also in brain development; thus, confirming Freud’s intuition regarding the central role of childhood and early experiences of the environment in our psychological development and our subjective outcomes. “Critical periods” of cerebral development represent temporal windows that mark favorable, but also circumscribed, moments in developmental cerebral plasticity. They also vary between different cortical areas. There are, therefore, strictly defined temporal periods for learning language, music, etc., after which this learning becomes more difficult, or even impossible, to acquire. Now, research into these critical periods can be seen as having a significant part to play in the interdisciplinary dialog between psychoanalysis and neurosciences with regard to the role of early experiences in the etiology of some psychopathological conditions. Research into the cellular and molecular mechanisms controlling the onset and end of these critical periods, notably controlled by the maturation of parvalbumin-expressing basket cells, have brought to light the presence of anomalies in the maturation of these neurons in patients with schizophrenia. Starting from these findings we propose revisiting the psychoanalytic theories on the etiology of psychosis from an interdisciplinary perspective. Our study works from the observation, common to both psychoanalysis and neurosciences, that experience leaves a trace; be it a “psychic” or a “synaptic” trace. Thus, we develop a hypothesis for an “absence of trace” in psychosis; reexamining psychosis through the prism of the biological theory of critical periods in plasticity.

## Introduction

Cerebral plasticity is our brain"s capacity to modify itself under the influence of our experiences that are influenced the environment. As early as 1909, the histologist Santiago Ramon y Cajal, who was opposed to the view of the nervous system as being static, had an intuition of this hypothesis “The nerve connections are thus not definitive and immutable, since associations are formed which are destined to subsist or be destroyed in accordance with undetermined circumstances.”(Ramón and Cajal, 1909). Since the 2000s, experimental data have brought to light molecular and cellular synaptic mechanisms of plasticity. These are mechanisms by means of which experience is inscribed in, and leaves a trace within, the neural networks. This modification of the nervous system due to the impact of interaction with the environment, is part of our organism’s process of adaptation and individuation. It is what makes the structure and functioning of our brain unique to each of us.

Synaptic plasticity of the brain remains possible throughout life, and into adulthood. It can also continue in physiological and pathological conditions, such as ageing. Until the 1990s it was deemed that adult neurogenesis (defined as the birth and development of new neurons in adulthood) did not take place after birth, but it has now been demonstrated that specific zones of the brain retain a neurogenic potential throughout life (Just et al., 2022).

However, there exists in the course of the individual’s development, precise temporal windows during which plasticity is at its peak. These temporal windows of plasticity, called *critical periods*, correspond to optimum moments when certain regions of the cerebral cortex can modify and remodel, both morphologically and physiologically, under the influence of environmental stimulations. When the critical period closes, this entails a loss of plasticity that makes the acquisition of new skills more difficult. This explains why, for example, it is easier to learn a second language or master a musical instrument during childhood rather that at adulthood.

The closing of critical periods, and the resultant partial loss of plasticity, can make the repair of damaged cortical pathways difficult. However, far from being only an inconvenience this closure is in fact essential for our development: once adulthood is reached, once the critical periods are closed, the cortex is less plastic, but it is also more stable. This make it possible to memorize and fix what has been learnt, to consolidate the traces resulting from our interactions with the environment that are the source of our autobiographical memories; something that contributes to anchoring our sense of identity. If our brain were to be too plastic, we would not be able to retain information, to consolidate the mnemic traces resulting from our past experiences. This situation could produce symptoms similar to those observed, for instance, in Alzheimer’s disease (Hensch, 2005; Testa et al., 2019).

The cellular and molecular mechanisms that are at the origin of the onset and closure of critical periods are beginning to be better understood. This promising new field of research is one that neurobiology has had a growing interest in for the last 10 years. A focus of this research is ultimately to shed new light on the role of critical periods when considering the impact of environment in some psychiatric disorders (Di Nardo et al., 2020; Vincent et al., 2021). This research, could amount to a significant point of convergence in a dialog between psychoanalysis and neuroscience in the field of mental disorders.

Freud had from the very beginnings of his work, emphasized the importance of interaction with the environment during childhood for psychological development, and the formation of personality (Freud, 1895, 1905). Similarly, he insisted on the role played by certain early experiences in the etiology of nervous disorders, observing that these might have “…more severe and lasting effects than they could do in mature years” (Freud, 1897). According to this view, the early part of an individual’s development could represent an important moment for understanding the impact of certain interaction with the environment on the individual’s mental and emotional development; including understanding the etiology of some mental illnesses. Freud also underlined the role of certain innate predispositions in the etiology of these pathologies (Freud, 1896). To understand the twofold influence of these innate factors, alongside factors acquired during childhood that can act in a complementary way, he put forward the idea of a series of associated factors that complement each other. According to this view of a multifactorial etiology of mental illnesses, pathologies might result either from: the combination of a strong innate disposition and some lesser acquired factors; of the presence of a minor innate disposition mixed with a strong incidence of events linked to interactions with the environment, during the critical period that stretches from the first months of life up to puberty; or, in some rare cases, a pathology could be caused by the influence of only one of these determining factors (Freud, 1917). Freud maintained this concept of a twofold etiology, looking beyond the alternative which would involve making a choice between exogenous and endogenous factors when determining the etiology of mental illnesses.

If we focus particularly on the etiology of schizophrenia, recent data from research in psychiatry seem to support, in a remarkable way, this hypothesis for a possible interaction between genetic and environmental factors. Initially studies into the origin of this pathology concentrated on searching for a genetic mutation (Riley et al., 2005). However, the hypothesis that schizophrenia is a single gene disorder has been abandoned for a number of years now (Brown, 2011; Ripke, 2014). This is because the development of genome-wide association studies (GWAS) has shown that vulnerability to schizophrenia is dependent on a large number of alleles with very small effects, and so can only partially explain the appearance of the schizophrenia phenotype (Sanders et al., 2008; Psychiatric GWAS Consortium Bipolar Disorder Working Group, 2011; Ripke, 2014; Misiak et al., 2018). Furthermore, these genetic factors are not specific to schizophrenia but can be found with other psychiatric pathologies (Van Snellenberg and de Candia, 2009; Hill et al., 2013; Skudlarski et al., 2013; Cardno and Owen, 2014; Wang et al., 2015; Misiak et al., 2018).

Studies of monozygotic twins have brought to light that, when one twin presents a phenotype for schizophrenia, there is a measure of agreement that goes from 40 to 60% of cases where the second twin presents the same phenotype (against a 10 to 15% for dizygotic twins; Fischer, 1971; Kringlen and Cramer, 1989; Cardno et al., 1999). This genetic matching is therefore relative, and the existence of a very high number of twins who share the same genetic heritage but where only one twin develops a phenotype for schizophrenia could be explained by environmental factors. Environmental factors can act *in utero*, and the fetal experiences of the monozygotic twins may prove to be different (Piontelli et al., 1999). They can also act during the perinatal period, during childhood, or even at adolescence (Brown, 2011).

Studies into the role played by the environment in the etiology of schizophrenia developed later than the initial research into the genetic etiology. This was due, among other things, to the methodological problems linked to these studies, as well as the difficulty in scientifically measuring the impact of environmental factors on the development of a phenotype for schizophrenia. Today however, we know that besides the genetic risks a number of environmental exposures can interfere with brain development, and originate some of the brain anomalies observed with schizophrenia. Among these factors, we find events that occur at different stages of development – and thus at critical periods involving distinct areas of the brain. In particular, we find: prenatal infections, obstetric complications, nutritional deficiency and *in utero* maternal stress, postnatal infections, trauma during childhood, consumption of toxic substances such as cannabis during adolescence, but also socio-economic factors such as growing up in an urban environment (Andreasson et al., 1987; Ames, 2001; Arseneault et al., 2002; Aleman et al., 2003; Allardyce et al., 2005; Dean and Murray, 2005; Amminger et al., 2007; Arion et al., 2007; Amminger et al., 2010; Brown, 2011; Marangoni et al., 2016).

Yet, when they are studied in isolation, these environmental factors, just as the genetic factors, present very low levels of effect and explain only partially the development of a vulnerability to schizophrenia. A 2017 study proposed a method (the polyenviromic risk score) for calculating the combined score of various environmental risk factors that seemed to corelate the most with a vulnerability for schizophrenia (Padmanabhan et al., 2017). However, insofar as genetic and environmental risk factors identified in schizophrenia remain of little significance taken in isolation, for a number of years now scientists have increasingly been focusing on the possible interactions between these two kinds of factors (Misiak et al., 2018) – in effect revisiting the Freudian hypothesis of a series of factors that complement each other, involving both types of determinants. Studies focusing on the interaction between these two factors aim to bring to light cases where the effects of certain environmental exposures are predominant in subjects who present certain underlying genotypes; or, alternatively, where the expression of a particular genotype would be dependent on the presence of given environmental factors (Ottman, 1996; van Os et al., 2008; Ayhan et al., 2016). The majority of studies focusing on gene/environment interactions have concentrated on the effect in variation of COMT, BDNF and FKBP5, which may interact with certain environmental exposures such as the presence of trauma or abuse in childhood, or the use of cannabis during adolescence (Brown, 2011; Misiak et al., 2018).

The research protocols for the link between genetic vulnerability and environmental exposure study three kinds of interaction (Clarke et al., 2009). One area of research is the increased effects of environmental exposure, owing to the presence of a certain susceptible genes (Brown, 2011). For example, the risk of schizophrenia developing in offspring of mothers who were exposed to pyelonephritis during pregnancy, is higher when antecedents for psychosis exist in the family and two genes (*DISC1* and *MIA*) may be involved. Another focus is the possibility that genetic vulnerabilities might constitute a risk factor for certain detrimental environmental exposures. This would be similar to the genetic predisposition to compulsive behaviors that can make some individual more susceptible to environmental incidents such as head trauma (Kendler and Eaves, 1986). Finally, there is research into the epigenetic factors involved in the etiology of schizophrenia. These studies look at functional changes that do not involve alterations in nucleotide sequence. They indicate that some environmental exposures that occur during critical periods in postnatal development can alter the epigenome, leading to changes in gene expression throughout the individual’s life (Zhang and Meaney, 2010). These alterations can be at the origin of an increased vulnerability for schizophrenia and, in some cases, are susceptible to transmission across generations (Brown, 2011).

Thus, some surprising similarities between recent data on the etiology of schizophrenia and some of Freud’s intuitions, have come to light. These similarities concern both the interaction between different factors for vulnerability, as well as the importance of critical periods in development during which the effects of certain environmental exposures are increased. If the idea of critical periods can represent a starting point for a promising dialog between psychoanalysis and the neurosciences, as regards research into psychiatric pathologies, some of the recent data in particular, encourages us to initiate that dialog. Indeed, as research in neurobiology has begun to better understand the molecular and cellular mechanisms that determine the onset and closure of critical periods, some studies have evidenced the existence of anomalies in the physiological mechanisms linked to critical periods of brain plasticity in schizophrenia patients.

The aim of our work is therefore, to initiate that dialog between psychoanalysis and the neurosciences around the role of critical periods in an understanding of schizophrenia, while at the same time, indicating the possible therapeutic horizons this interdisciplinary approach presents. To that end, we propose first to define the biological concept of *critical periods*, using the model of a developmental pathology linked to the acquisition of binocular vision, namely amblyopia. This pathology has allowed scientists to better understand the molecular and cellular mechanisms that determine the onset and closure of critical periods. We propose describing some of the mechanisms linked to the maturation of inhibitory parvalbumin-expressing interneurons, and we will discuss the anomalies related to the functioning of these inhibitory cells that have been observed in schizophrenia patients.

We will then revisit the research around the cellular mechanisms of critical periods and their anomalies observed in schizophrenia, through the prism of a dialog between psychoanalysis and neuroscience. This dialog is based on the intersection of these two disciplines around the concept of “trace” (Ansermet et al., 2007). This model draws on the fact that psychoanalysis and neuroscience come together around the shared observation that experience is inscribed leaving an organic trace. On the basis of this model, we will endeavor to reframe the Freudian and Lacanian psychoanalytic theories on the etiology of schizophrenia – thus putting them into perspective with the research done on critical periods and the amblyopia model. We propose putting forward an interdisciplinary view of schizophrenia as resulting from an “absence of trace,” that is to say, a defect in inscription of certain experiences linked to interactions with the environment during critical periods of brain plasticity. We will then propose some hypotheses relating to the nature of these traces – traces that may be at the root of our sense of self, and of identity –, as well as the effects resulting from the absence of their inscription (or pathological inscription, as is the case in amblyopia) for schizophrenia. Finally, we will consider, from an interdisciplinary perspective, the therapeutic possibilities offered by the work being done around the reopening of critical periods for plasticity.

## Critical periods as temporal limits to plasticity: The amblyopia model

The research done by David Hubel and Torsten Wiesel on the visual system of cats – for which they received the 1981 Nobel Prize in Physiology or Medicine – was the first to experimentally demonstrate the temporally circumscribed character of cerebral plasticity. Their questions stemmed initially from a disease named amblyopia. In humans, amblyopia is a pathology characterized by a unilateral visual impairment in children who had been operated too late for cataracts, or who presented a significant strabismus that was not treated before the ages of four or five. The absence of stimulation in one eye during a certain length of time, led to a definitive weakening of cortical representation in the visual cortex for that eye in favor of the visual field of the other eye (Prochiantz, 2012). Although the term commonly used for this disorder is “lazy eye,” it is however not a lesion or a functional disorder of the eye that is the root cause. The sensory organ itself is completely functional, it is at the cortical level that the representation is absent (the representation of both eyes is modified). Wiesel describes having initially tried to build an animal model that could demonstrate this particularly of the visual system"s development, based on experimentation using kittens. The results brought to light that, depending on the timing and the duration of the suture of one eye, the development of the visual system in the visual cortex showed noticeable differences. If the sutures were performed immediately after birth and left for 3 months, the kitten were definitively blind in the eye that had been closed (Wiesel, 1982). Even once the eye was allowed to open, the majority of neurons situated in the visual cortex only respond to stimulations from the eye that had remained open. The work of Hubel and Wiesel, thus made it possible to demonstrate experimentally that the development of binocular vision requires the stimulation of both eyes, during a very specific timeframe in postnatal development. Their research showed that the development of the visual cortex is dependent on the environment. The absence of stimulation in either of the two eyes, can have irreversible consequences for the acquisition of binocular vision.

If a kitten’s eye is closed 2 weeks after birth, at the end of 18 months only the eye that was left open will give rise to cortical activity. However, if the eye is closed 10 weeks after birth, the impact is lessened, and significant activity can be observed in the cortical region when stimuli is applied to that eye. Closing of the eye that occurs after 1 year still shows some impact, but far less than the two previous interventions; and if the closing takes place at 6 years of age, no modification or reorganization of the cortical circuits are observed (Prochiantz, 2014; Di Nardo et al., 2018; Di Nardo et al., 2020). There exists therefore a precise temporal limit for the establishment of the phenomena of brain plasticity, insofar as that it is significantly reduced after a certain “critical” period for the development of any given brain function (in this instance binocular vision). After the closure of the critical period within the primary visual area, treatment of amblyopia becomes more difficult, even impossible (Scheiman et al., 2005). Sensory experience therefore, plays a significant role in the building of the central nervous system. The work of Hubel and Wiesel contributed to showing that, although brain development is in part determined by innate genetic factors, that development can be very labile within any given genetic makeup. The environment, especially through certain early experiences, modifies in a durable or even irreversible way the structure of the nervous system. These modifications can, however, only happen if the stimuli occur within a precise temporal window. Brain plasticity is therefore not limitless throughout development, and interactions with the environment will not have the same effects on the organism depending on when they take place (Hensch, 2005). Consequently, there exist strict temporal windows that limit the impact of experiences on the organism and on the nervous system.

If the work done on critical periods within the nervous system has played an important role in the experimental demonstration of this phenomenon, the existence of such temporal windows for the development of the central nervous system affects many other areas of the brain, and the critical periods for these different areas of the cortex are not synchronous. Depending on the regions of the brain and the circuits involved, the critical periods can occur at very different stages of overall development. In the case of some functions, plasticity persists up to quite a late age such as adolescence. While other faculties have a plasticity that is limited to the first years of infancy.

There are critical periods for various processes of increasing complexity, and they generally happen in a cascade through different neuronal circuits (Di Nardo et al., 2020). Initially they occur within the primary sensory areas such as sight or hearing; then they spread to reach the parts of the brain that handle motricity and language. The physiologist Jeannet Werker evidenced a hierarchy of these critical periods within the different cortical areas that are involved in language, and the acquisition of the ability to recognize the maternal language and its pronunciation. Each area has its own window of plasticity (Werker and Hensch, 2015). Then, much later, a critical period takes place in the areas linked to the control of emotions and cognitive functions; these remain able to modify up to the end of adolescence (Takesian and Hensch, 2013; Werker and Hensch, 2015; Nelson III and Gabard-Durnam, 2020). Additionally, depending to the areas that underpin these different skill acquisitions, the duration of the critical periods appears to be vary widely: from a few months for the ability to discriminate phonemes for example (and other sensorial areas), up to several years for higher cognitive functions. As an example, the prefrontal cortex has a neuronal circuit that has a particularly lengthy period of plasticity that continues up until puberty and also in young adults.

## The cellular mechanisms behind critical periods: The role of parvalbumin-expressing inhibitory neurons

Despite the diversity of cerebral areas that are subject to critical periods, studies on mice have brought to light that the physiological mechanisms that control the onset and closure of these periods are the same (Di Nardo et al., 2020). Furthermore, these mechanisms are closely linked to the maturation of inhibitory neuronal circuits. The balance between excitation and inhibition within the cerebral cortex is key to understanding the physiological mechanisms that regulate critical periods (Hensch, 2005; Bernard and Prochiantz, 2016). Inside the nervous system, it is the levels of inhibitory activity of particular GABAergic neurons that plays a determining role in the balance between excitation/inhibition. Their inhibitory neurotransmitter is Gamma-aminobutyric acid (GABA), which when liberated inhibits the firing of the postsynaptic excitatory neurons. The onset of critical periods occurs at the start of the maturation of a particular group of inhibitory GABAergic neurons called *basket cells*, which synthesis a specific protein called parvalbumin (PV). Experiments performed on the visual cortex of mice show that the critical period closes when these neurons reach complete maturity (Hensch, 2005; Werker and Hensch, 2015). Thus, the onset of a critical period is triggered by the maturation of the PV expressing inhibitory interneurons, and when maturation reaches its peak the period closes, resulting in a considerable decrease in the plasticity of the circuit. Consequently, when inhibition reaches a certain level the circuit becomes closed, losing its plasticity. It can be observed that, at the moment of their maturation, the basket neurons physically become “fixed” by surrounding themselves with perineuronal nets (PNNs). These PNNs are an extracellular matrix structure, in the form of a proteinaceous net enriched with complex sugars from the glycosaminoglycan family (GAG). These structures contribute to stabilizing the system and considerably reducing its plasticity (Hensch, 2005; Bernard and Prochiantz, 2016; Testa et al., 2019; Di Nardo et al., 2020). Additionally, the balance between excitation and inhibition that is reached at the end of the critical period, through the maturation of the PV inhibitory neurons, is necessary to maintain the non-plastic state of the adult brain. That inhibition reduces the plasticity in the adult brain, is demonstrated by the increase in plasticity that follows the reduction in GABAergic transmission in adult rodents, caused either by a GABA antagonist or by the inhibition of GABA synthesis (Harauzov et al., 2010).

Scientists have noted that, from the point of view of energy, the metabolism of PV producing cells involves a significant and sustained physiological activity (Alitto and Dan, 2010). Indeed the GABA released by the PV cells binds to the alpha-1 type GABAergic receptors present on the cell body of the pyramidal neurons. Owing to the rapid desensitization of the receptors and the excitatory feedback loop coming from the postsynaptic cells, a high-frequency (between 40 Hz and 80 Hz) oscillation occurs during the critical period when inhibition is at its peak. The release and capture of ions as the depolarization and repolarization of the membrane of the PV cells takes place requires the production of high levels of ATP. The metabolic rate of the PV cells is thus very high, and a deficient metabolism can therefore have serious physiological consequences on the functioning of these inhibitory circuits (Prochiantz, 2014).

A study on primates undertaken in 1996 showed that the maturation of PV cells took place at an earlier stage in the sensory areas A1 and V1, and progressively later, depending on its progress, through the hierarchy of the cortical areas (Condé et al., 1996). In humans, the critical periods for discriminating phenomena or for the acquisition of binocular sight also end early on, since the sensorial neuronal networks become fixed relatively early on in the course of development. However, it should be borne in mind that it is the photons hitting the retina or the waves hitting the internal ear cilia that allow for the initial maturation of PV cells. There is no clock for this maturation, and sensorial deprivation, as keeping both eyes closed in animals, delays PV cell maturation: plasticity does not open until the eyes are open. This also explains why the critical periods are not synchronous (Werker and Hensch, 2015).

In contrast to the sensory areas, the neuronal networks of the prefrontal cortex, associated with higher cognitive functions and mood regulation, have a particularly long period of plasticity: the maturation of PV cells is observed in these areas only at the onset of puberty, with a critical period that ends around the age of 20 years. The exceptionally slow nature of the maturation of the PV cells within these cortical areas is especially noticeable in humans (Prochiantz, 2019).

## The role of OTX2 in critical periods and their environmental modulation

Among the factors identified that regulate the maturation of the PV neurons, the homeoprotein OTX2 plays a key role. It is a transcription factor, that is, a protein which is necessary for regulating the transcription of certain genes, while also interacting with the DNA and RNA. The homeoprotein OTX2 is transferred from an extra-cortical source (the choroid plexus) to the cortex (Spatazza et al., 2013; Di Nardo et al., 2020). An initial activity-dependent condensation of the extracellular matrix of PV cells into perineuronal nets (PNNs) allows OTX2 internalization, initiates the opening of critical periods at a first concentration threshold and closes it at a second one. OTX2, once internalized, maintains PNN assembly, initiating a positive loop of interaction between PNNs and OTX2 (Sugiyama et al., 2008). This mechanism first demonstrated in the mouse visual cortex (V1) was generalized to the primary auditory cortex (A1), and medial prefrontal cortex (mPFC). Lee et al., 2017). Since, choroid plexus-derived OTX2 accumulates in PV cells throughout the cerebral cortex, it can be proposed that it is a general critical period regulator.

As mentioned above, the regulation of the onset and closure of critical periods through the accumulation of OTX2 takes place within a process that involves a two-thresholds model (Di Nardo et al., 2020). At the first OTX2 accumulation threshold, the window of plasticity opens. Then, the crossing of a second threshold of accumulation triggers a closure of the plasticity and maintains the non-plastic state of the adult brain. Prochiantz and Di Nardo, 2015). In the experimental protocols put in place using mice, a cortical infusion of the OTX2 protein accelerates the appearance and the closure of the critical period. While if OTX2 internalization by PV cells is blocked in a region of the cortex, the onset of the critical period is delayed in that region. Most importantly, permanent OTX2 import is necessary to maintain the adult non-plastic state and, as shown in V1, decreasing OTX2 in adult PV cells temporarily reopens plasticity.

Thus, if the influence of OTX2 on the maturation of PV cells has a direct incidence on the onset and closure of the critical periods, genetic or environmental factors that act upon the expression of OTX2 or its assimilation by the PV cells can result in a poor synchronization of the critical periods for plasticity within the cortex, possibly resulting in subsequent disorders. Genetic modelling in mice has shown that a genetic point mutation in the recognition motif of the GAG PNN by OTX2 delays the maturation of the PV interneurons not only in the primary visual area, but also in the primary auditory area, and the mPFC. This delayed plasticity can be correlated with a reduction of anxious-like behavior in mice (Lee et al., 2017). And more recently Vincent et al. have demonstrated that anxiety-like behaviors in the adult can me modulated by changing OTX2 levels in the choroid plexus or blocking its transfer into mPFC PV cells (Vincent et al., 2021). OTX2 signaling, and the synchronization of critical periods, can also orchestrate complex behaviors, reflecting the interaction of several sequential critical periods, as with language (Werker and Hensch, 2015). In consequence, a disrupted OTX2 signaling may be the cause of the onset of certain psychiatric and cognitive disorders (Gogolla et al., 2009; Le Magueresse and Monyer, 2013; Maeda, 2015).

Besides these genetic factors, environmental factors can also act upon the OTX2 signals, and influence the onset and closure of critical periods. In dark-reared mice, the retina is not stimulated and the activity signal is not sent to the cortex (Sugiyama et al., 2008). Thus, PV cells are not informed that the eyes are opened (because of the dark) and do not start to assemble the PNNs. As a consequence, OTX2 is not captured by the PV cells. In this mice, a 70% decrease in the quantity of OTX2 present in the PV cells was observed (Sugiyama et al., 2008). This absence of OTX2 capture does allow a further reinforcement of PNN assembly. The initial PNN assembly is activity dependent - if not all critical periods would be synchronous. It appears then, that the onset of critical periods requires adequate environmental stimuli to regulate the OTX2 signals (Di Nardo et al., 2020). Another study on mice has demonstrated that depriving the newborn of the maternal presence during a critical periods stretching from the 10^th^ to the 20^th^ day postpartum can result in a higher percentage of the offspring being subject to permanent anxiety and/or depression – even if some individual appear to present a degree of resilience (Peña et al., 2017). In the non-resilient progeny, the expression of OTX2 is transiently weaker in the ventral tegmental area (VTA) during the period of maternal separation. We should recall that the VTA is a dopaminergic hub in the mesencephalon, whose neurons project into the cortex and the sub-cortical structures, with a greater number of projections in the limbic area. The role of OTX2 in complex traits such as depression or anxiety has been evidenced not only in murine models of early stress, but also in humans, where studies have been done with children victims of maltreatment (Peña et al., 2017; Murthy et al., 2019; Vincent et al., 2021). Samples of DNA from children who had suffered maltreatment showed a correlation between the presence of depression and the state of OTX2 gene methylation, and of genes downregulated by OTX2 (Kaufman et al., 2018). This would seem to strengthen the hypothesis that OTX2 is an important modulator of mental health (Di Nardo et al., 2020).

## Cellular anomalies linked to critical periods in schizophrenia

Anomalies in the maturation of PV cells correlate with a number of psychiatric disorders (Gogolla et al., 2009; Lee et al., 2017); (Maeda, 2015). To explain these anomalies, an OTX2 hypothesis can be envisaged but is not the only one, and other models are not incompatibles with a regulation by OTX2, like the oxidative stress model which also explains PV cell maturation and excitation/inhibition balance modifications in schizophrenia. For example, the dysfunction of PV cells can be explained by the high metabolic requirements of these cells, which may render them susceptible to redox dysregulation and oxidative stress. A protocols using mice carrying a genetic redox imbalance has demonstrate that PNN play a critical role in the protection against oxidative stress. Although the perineuronal nets act as a protective shield, they are also themselves sensitive to excess oxidative stress. The protection might therefore reflect a balance between the oxidative burden on perineuronal net degradation and the capacity of the system to maintain the nets. Abnormal perineuronal nets, as observed in the postmortem patient brain, may thus underlie the vulnerability and functional impairment of pivotal inhibitory circuits in schizophrenia (Berretta et al., 2015).

More generally, it has been observed that patients with schizophrenia present a lower density of PNNs than control groups, within the amygdala, the entorhinal cortex, and the dorsolateral prefrontal cortex (Pantazopoulos et al., 2010; Mauney et al., 2013; Berretta et al., 2015). This translates into an anomalous maturation of the PV cells in this region. The prefrontal cortex is one of the areas of the cortex whose disfunctions have been particularly associated with schizophrenia (Callicott et al., 2000). In this region of the brain, in healthy subjects, the maturation of the PV neurons at puberty usually triggers an increase in the quantity of PNNs, which translates into an increase in inhibition. In schizophrenia patients, although the density of PV neurons is not abnormal, it is a fault in their maturation that creates a disruption in inhibition within the prefrontal cortex (Enwright III et al., 2018). Hence the reduction in high-frequency gamma oscillations, and a lower PNN density observed in some patients (Uhlhaas and Singer, 2015; Di Nardo et al., 2020). Lacunae in the number of inhibitory synapses can also be observed in schizophrenia patients, along with a reduction in the pruning of excitatory pyramidal neuron dendrites (Insel, 2010).

It is therefore an increase in excitation, to the detriment of inhibition (a modification in E/I balance), that takes place within the neuronal circuits. Following this idea, scientists have noted an immaturity of the PV cells within the prefrontal cortex of schizophrenia patients (Mauney et al., 2013). These results have been confirmed by Daniel W. Chung’s team, who have also brought to light a significant reduction in the expression of PV within the dorsolateral prefrontal cortex of schizophrenia patients (Chung et al., 2018). There could therefore be a link between this kind of psychiatric pathology and an anomaly in the timing of the periods of plasticity. Such periods of plasticity would extend over an abnormal length of time, owing to a lack of inhibition, subsequent to a late maturation of the inhibitory circuits (Mauney et al., 2013). Still, it is difficult to determine if these alterations in the balance between excitation-inhibition and the gamma oscillations in schizophrenia, linked with the faulty maturation of the PV neurons that do not sufficiently surround themselves with PNNs, could be a cause or a consequence of the pathology (Testa et al., 2019).

To bring some answer to this question, it is appropriate to note that certain genetic etiological factors and environmental factor associated with schizophrenia, can have consequences for the maturation of the PV cells and their PNNs. For example, several studies highlight a link between schizophrenia and anomalies in the expression of certain genes that are necessary for the formation of the PNNs (such as NEUROCAN, HAPLN4, or PTPR; Schizophrenia Working Group of the Psychiatric Genomics Consortium, 2014; Testa et al., 2019). Similarly, murine models that use environmental exposure to induce a phenotype that mimics aspects of mood disorders in mice, show anomalies in the maturation of the PV cells. For example, a study in mice using maternal immune activation during pregnancy to induce anxiety-related phenotypes among the offspring, has shown that these mice present a reduction of PNNs in the mPFC and the amygdala (Paylor et al., 2016). Similarly, as we have already observed, in mice the exposure to early stress in the first day of life induces changes in the expression of OTX2 in the VTA or the choroid plexus, and it is known that the expression of OTX2 in the choroid plexus regulates the maturation of PV neurons (Peña et al., 2017; Di Nardo et al., 2020; Vincent et al., 2021).

The alterations in the PNNs observed in schizophrenia patients could therefore appear as a consequence, linked with the genetic and/or environmental factors associated with schizophrenia. However, it should also be pointed out that the removal of the PNNs in the hippocampus of mice can also directly bring about behaviors similar to the positive symptoms of schizophrenia (Shah et al., 2013). Likewise, experimentally inducing a lowering of the transfer of OTX2 from the choroid plexus to the PV neurons in rodents, results in anxiety-related phenotypes (Vincent et al., 2021). The lack of inhibition linked to the abnormalities in the maturation of the PV neurons that regulate critical periods could therefore, in the case of schizophrenia, be associated with an excess of plasticity. Such an excess of plasticity, is at the root of certain symptoms observed in this pathology.

But if we concentrate on a defective maturation of PV cells on the basis of markers, maturation could be so low that the first threshold (plasticity opening) is not even passed. Then the schizophrenia phenotype would be a consequence of an absence of plasticity when needed to repose to an environmental change.

Yet, if this faulty maturation of the PV interneurons has been recognized as associated with the disorders in cerebral development present with schizophrenia, this observation is compatible with an interaction of genetics and environmental factors in schizophrenia etiological models (Prochiantz, 2019).

## A dialog between psychoanalysis and neuroscience around the anomalies in critical periods observable with schizophrenia: The hypothesis of the absence of trace

Even if much remains to be done, and caution should be exercised, recent discoveries about the cellular and molecular mechanisms determining the onset and closure of the critical periods of plasticity have opened a new field of research in the understanding of certain psychiatric disorders such as schizophrenia. If the anomalies in the maturation of PV cells within the prefrontal cortex observed in this pathology can be both genetic and environmental in origin, their primary consequence is a disorder in the timeframe of critical periods for plasticity. This disorder can impact on the manner in which the environment models and structures the development of the cerebral cortex. Indeed, if the onset of a critical period does not occur at the right time, or if stimulations from the environment do not take place in a suitable way at the time when the critical period is open (as was exemplified with the case of amblyopia), the connections of the neuronal networks can fail to be put in place, or be inadequately put in place, within the cortex. In other words, for the stimuli linked to our interaction with the environment to be inscribed within our nervous system and guarantee a good development of our neuronal connections, these stimulations need to take place at the moment when the plasticity is at its peak, during the critical period for the region of brain concerned, and then be stabilized and consolidated through the closure of the window of plasticity. When it has not been possible for the environmental experiences to be inscribed in the form of neuronal traces during the critical period of plasticity for a given region of the cortex, one of the consequences is that we observe anomalies in the structure of the cerebral cortex in adulthood. This can be seen in the data from brain imaging of patients with amblyopia. Now, if anomalies linked to the maturation of the PV cells in schizophrenia cause either a delay in the onset of a critical period, or a failure in its closure, the consequence may be that certain traces resulting from interaction with the environment might not be inscribed, or would not be adequately consolidated in the cortical regions involved, including the dorsolateral prefrontal cortex. This hypothesis coincides with the data coming from brain imagery of patients with schizophrenia, where numerous anomalies in the structure of the cortex can be observed (Arnone et al., 2009; Gur and Gur, 2010).

Structural imaging data have shown that schizophrenic patients tend to have a thinner cortex in parts of the brain such as the insula, have enlarged lateral ventricles, and have smaller hippocampal volumes (Haukvik et al., 2013). With regard to functional imaging data, many brain regions and networks show abnormal connectivity in schizophrenia. The principal results observed concern a hyperactivity of the default mode network (Hu et al., 2017) and functional connectivity anomalies in the insula (Wylie and Tregellas, 2010), hippocampus and prefrontal cortex (Lalousis et al., 2022). Aberrations observed on functional MRI scans in these regions are particularly interesting to relate to the PV cells’ abnormalities. The significant loss of PV cells in the hippocampus can lead to hyperactivity of this region that then induces a hyperdopaminergic disorder. This is associated with psychotic symptomatology. Data on the hyperactivity of the dopaminergic system in schizophrenia could thus appear to be strongly correlated with the loss of PV cells in the ventral hippocampus (Grace and Gomes, 2019). A recent study has proposed an animal model of stress during critical periods, to explain abnormalities of the hippocampus and prefrontal cortex in schizophrenia. Exposure to a prolonged level of stress impacts the ventral hippocampus, inducing an abnormality in PV cell function and cell loss. This contributes to the hyperactivity of glutamatergic pyramidal neurons. Therefore, a hyperdopaminergic state appears – which is strongly associated by many studies with psychotic symptomatology. Furthermore, the medial prefrontal cortex, and particularly the prelimbic portion, has been shown to be involved in the control of stress response in basolateral amygdala. The implication is that the prefrontal cortex would no longer be able to regulate the amygdala’s reactivity to stress. This would then lead to glutamatergic hyperactivity, which in turn could induce damage to parvalbumin cells and then generate a hyperdopaminergic state (Gomes et al., 2019).

If the latest advances in biology research on critical periods show promise for the understanding of the role of early interactions with the environment in the etiology of schizophrenia symptoms, these discoveries also open the way for an innovative dialog with psychoanalysis. Freud also had very early on emphasized the important place held by our childhood experiences, and early stimuli linked to the environment, in our subjective construction, as well as in the appearance of certain psychopathological disorders (Freud and Breuer, 1895; Freud, 1905). The work of several post-Freudian psychoanalysts, such as Jacques Lacan, also sought to apply this perspective to the understanding of the psychopathology of psychosis (Lacan, 1997). To draw a comparison between these psychoanalytic hypotheses and research in neurobiology on the critical periods, we are now going to propose integrating this research into a previously formulated model of interdisciplinary dialog (Ansermet et al., 2007; Ansermet and Magistretti, 2010; Tran The, 2022).

This model of dialog between psychoanalysis and neuroscience is rooted in the fact that both these disciplines, despite their radically different epistemological foundations, nevertheless meet around the idea of “trace.” Indeed, if the neurosciences have demonstrated that experiences stemming from our interactions with the environment are inscribed in the form of traces (such as synaptic traces) within the nervous system – traces that integrate structural and functional modifications in the organization of neuronal networks –, the concept of “trace” is also at the root of the whole Freudian theory of the functioning of the psychic apparatus. In this respect, psychoanalysis and neuroscience come together over the shared observation that experiences linked to the environment are inscribed and leave a trace, whether we think of this trace as “physical” in the neurosciences, or as a “psychic” trace in the psychoanalytic perspective (Freud, 1887-1904).

This model is also based on the fact that, if experiences are inscribed and leave a trace (both psychic and synaptic), these traces are associated with somatic states. Representations (*R*) resulting from exteroceptive perceptions are linked, owing to an association through simultaneity, with representations resulting from interoceptive perceptions of somatic states (*S*) that have been the object of a concomitant and synchronic perception. Experimental discoveries around synaptic plasticity and the neurobiological mechanisms of memory, as well as the Freudian theory of the formation of the first mnesic traces in the experience of satisfaction, put the emphasis not only on the perceptions stemming from the external environment, but also on the paramount importance of the interoceptive perception of the organism’s state (be it a corporal perception of pleasure or displeasure) that accompanies the external perception (Freud, 1900). The action of the perception of the internal states of the body are therefore critical to the inscription of the experience; and the simultaneity of the perceptions *R* and *S* appear to be an essential condition for the inscription of the trace in the form of an association *R-S*.

Furthermore, the Freudian definition of thought as being in the service of the pleasure principle, Freud (1900) as well as the biological definitions of thought proposed by neuroscientist such as Alain Prochiantz – who sees thought as “an adaptive relationship that binds the individual and the species to their environment” [our translation](Prochiantz, 2001) –, or Antonio Damasio, according to whom the human faculty of reasoning appeared in evolution primarily to serve homeostasis, Damasio (2017) all these theories imply seeing cognitive function withing a homeostatic framework where the representations produced by our thoughts fulfil the purpose of regulating our somatic states. On the basis of this coming together of psychoanalysis and neuroscience around a homeostatic understanding of the psychic functioning (and in particular thought processes), we can underline the importance of this homeostatic functioning of the trace. This function appears to be a point of intersection between these two field, whose epistemological foundations are yet incommensurable (Ansermet et al., 2007).

In his model of the newborn’s experience of satisfaction, Freud described how the first experiences linked to interactions with the environment – most notable during the fulfilment of the newborn’s basic needs by the adults who care for it – were inscribed within the psychic apparatus (Freud, 1900). The exteroceptive perceptions – that accompanied the pleasure that results from the satisfaction of basic needs such as hunger – are inscribed in the infant’s memory, and these mnesic traces that are the origin of the first representations, play the role of regulators in the infant’s first somatic experiences. These first experiences are marked by a state of complete helplessness, which Freud termed *Hilflosigkeit*. This is a state in which “unpleasure” dominates the first corporal experiences of the newborn. According to the psychoanalytic perspective, in the child’s subsequent development, different traces (notably the Oedipus complex) that stem from interactions with the environment, as well as those produced by the subject themselves, perform this homeostatic function.

It is possible to integrate the Freudian model of the experience of satisfaction, and neuroscientific research on the role of the perception of somatic states in the inscription of experiences (Tran The, 2022). For example this has been done in research on interoception,(Craig, 2009) or on somatic markers (Damasio, 1996). In this interdisciplinary perspective, the representations (R), which come from the environment, from language and from those who take care of the newborn, play a regulatory role on the body states and on the interoceptive perceptions (S). This perspective also accords with Wilfred Bion’s description of the alpha function of the psychical apparatus. The child is invaded by “beta” content, such as somatic perceptions that are sources of anxiety, in a raw relationship to the reality of its body and the world. The alpha function corresponds to the subject’s capacity, through the mediation enabled by the adult, to metabolize beta elements. According to Bion, the prototype of the alpha function is the parent’s capacity for dreaming. The parent takes into himself the contents deposited by the infant and restores elaborated alpha elements to the infant. In turn, these elements then allow the infant to distance himself from experiences, and from the intense and painful sensations resulting from its state of *Hilflosigkeit*.

Another prolongation of the Freudian understanding of trace inscription resulting from the newborn’s experience of satisfaction, is the perspective put forward by Lacan. Based on Saussurean linguistics and Lévi-Strauss’ structural anthropology, the psychoanalyst Lacan proposed rereading the Freudian concept of “*trace*” in term of “*signifier*.” Lacan’s argument was that “The memory phenomena that Freud is interested in are always language phenomena” (Lacan, 1997), and language is the result of the incidence of intersubjective relationships with others – including the adults caring for the child during its development. Thus, Lacan draws the field of the signifier into that of the “Other,” which is to say alterity or otherness in its language dimension. From there he will postulate the existence of a field of the “primordial signifier,” of traces whose function is homeostatic and structuring for psychic development (Lacan, 1997). Based on this idea, the Lacanian psychoanalytic perspective understands psychosis as the result of an absence of inscription in childhood of some of those traces essential to our subjective construction. Thus, by rereading Feud’s theory on the organizational role of the Oedipus complex in psychic development, Lacan proposed the hypothesis of an etiology of psychosis based on the signifier (Lacan, 2002a). Lacan develops his theory of “*foreclosure*,” that is to say the rejection of the primordial signifier, as a precondition for psychosis (Lacan, 1997). In line with this position, at the origin of psychosis one would find the absence of inscription of what is, for the psychic apparatus, a primordial and structuring representation – be it the unifying of the image of the body or the organizing role of the Oedipus complex (Lacan, 2002a).

The Lacanian psychoanalytic hypothesis, reformulated within the framework of the model of an “*R-S”* interdisciplinary dialog, is in essence to see psychosis as being characterized by the absence of, or defective inscription of, certain traces whose function is homeostatic and structuring for our psychic development. This hypothesis of an absence of trace, which stems from a psychoanalytic theory of psychosis, offers an ideal formalization for an interdisciplinary dialog, since it draws on the paradigm of the trace that is common to neuroscience and psychoanalysis. Thus it forms a possible intersection between these two disciplines.

Research in neurobiology on the cellular and molecular mechanism of critical periods for cerebral plasticity, which shed light on the role of early interactions with the environment in certain psychiatric pathologies like schizophrenia, resonates with this psychoanalytic idea of an absence of traces in psychosis. Research on the critical periods for plasticity have demonstrated that the inscription of traces stemming from experiences with the environment is conditioned by various factors. Obviously, it is necessary that the interaction does take place – for example, in the case of the visual cortex, it is necessary that an effective environmental stimulation of the eyes is produced for the neural connections to be put in place. However, it is also essential that this interaction with the environment takes place during a precise temporal period – a timeframe situated between the moment of the onset of the critical period of plasticity for the region of the brain in question, and its closure.

The discovery in schizophrenia patients of certain anomalies at the level of the molecular and cellular mechanisms that determine the onset and closure of critical periods, represents a major area for discussion in the dialog with psychoanalysis regarding the absence of traces as the origin of psychosis; since critical periods play such a determining role in the inscription of early experiences through the mechanisms of plasticity. On that basis, we can envisage that the inscription of the first representations *R* that stem from exteroceptive perceptions (associated with the interoceptive perceptions of corporal states *S*) whose function is homeostasis, could only take place within a limited timeframe defined by a critical period. Thus, we can postulate that in psychosis the inscription of traces whose functions are structuring for psychic development, may not have taken place within that limited timeframe – either because the critical period was not open at the time of the interactions with the environment, or because the environmental stimulations did not occur at the appropriate time (as in the amblyopia example).

Different clinical models can be used to illustrate how breakdowns in the relationship with the environment can result in an absence in the inscription of traces that structure psychic development. Levine’s study demonstrated that, confronted with a prolonged period of maternal separation in their early development, young rodents initially presented anxious and agitated behavior, but that subsequently significant manifestations of behavior similar to despair appeared. These were accompanied by important physiological changes. The body temperature of the young rats dropped significantly, as did their heart rate. This resulted in a degree of withdrawal and a lessening of motricity amounting to a kind of catatonia (Levine, 1957). A parallel can be drawn between these observations and research done in humans on the effects of family separation during the Second World War in the U.S. – notably the syndrome of hospitalism described by Spitz. This study highlights the psychic, cognitive and physiological consequences of emotional deprivation in children who were separated from family and raised as orphans (Spitz, 1946). A parallel can be drawn with the study done by Nelson that appeared in *Nature* in 2007 following the “Bucharest Early Intervention Project (Nelson III et al., 2007). The latter study concerned children raised as orphans during the Ceausescu regime in Romania, under extremely precarious circumstances from a emotional and material perspective. It showed that children who were place in foster families sufficiently early, demonstrated significant advantages in terms of emotional, cognitive and social development in comparison to those children who were placed only after 20 months. This occurred despite the two groups of children (raised in institutions or raised in foster families) both presented anomalies in the volume of gray matter when compared with children in a control group who had not experienced family separation.

These works are interesting for showing the psychic, cognitive and cerebral consequences of the absence of certain stimulating environmental interactions in the first months of infant development. The hypothesis for anomalies in critical periods in schizophrenia allows for a shift in relation to a direct linear causal view between certain environmental experiences and the psychopathological consequences. It should be underlined that an understanding of the molecular and cellular mechanisms for the onset of critical periods demonstrates that, even in the event of an appropriate and stimulating environment, neurobiological anomalies could have the same consequences for the brain and psyche as the absence of stimulation. It is from this pluri-factorial perspective, and not from a linear one, that the interdisciplinary hypothesis for the absence of trace in schizophrenia stems.

## The onset of schizophrenia in adolescence as a consequence of the absence of trace

The hypothesis of a link between the physiology of critical periods and certain psychiatric pathologies has become a new field of research for neurobiology, notably in view of the delayed maturation of PV cells in the prefrontal cortex that has been observed. This late maturation, occurring at puberty, could be correlated with the generally accepted fact that the onset of schizophrenia typically occurs during puberty (Prochiantz, 2012).

From the psychoanalytic perspective, the triggering of psychosis is understood as a two-phase process. This process involves firstly that, during childhood, certain traces whose function is homeostatic and structuring for psychic development have not been inscribed in the subject. However, for the symptomatology to appear and for psychosis to be triggered in a manifest way, certain life events in the subject’s trajectory need to solicit those missing traces (Lacan, 2002a). In as much as those traces concern, among other things, the Oedipal question – that is to say a relational complex with the parental figures, involving strong affective elements, as well as questions of origin, of reproduction, sexual identity and the difference of the sexes – all event that solicit these themes can lead to the triggering of psychosis (Lacan, 2002). Puberty, which raises the question of sexual identity and identifications inherited from the Oedipus complex, is a key moment for the revival of the Oedipal problematic. Therefore, this juncture can turn out to be conducive to soliciting those missing traces (or what Lacan terms “foreclosed signifier”), leaving the subject facing emptiness and an absence of meaning that prevents them from responding to the reconfigurations that take place at puberty (Lacan, 2002a).

From a biological point of view the physiological changes, notably hormonal changes, during puberty, also demand a process of adaptation and a significant reconfiguration in the relationship to the environment. As Prochiantz points out, in this regard schizophrenia could be linked to “an inability on the part of the brain, be it for genetic or developmental and epigenetic reasons – both are possible and can combine –, to mobilize the necessary plasticity for that adaptation” [our translation] (Prochiantz, 2012).

Although the disorder may already have an underlying presence before puberty – be it genetically determined, or epigenetically determined by early environmental interactions – puberty and the transition between the end of adolescence and the beginning of adulthood would seem to be a particularly favorable moment for the appearance of the symptomatology of schizophrenia; that is to say, for the expression of the phenotype that characterizes this pathology. One of the neurobiological hypotheses is that there is a link between the decompensations frequently observed at adolescence with schizophrenia patients, and the maturation of the PV cells in the dorsolateral prefrontal cortex that takes place only at the end of adolescence (Prochiantz, 2014). The hypothesis is that PV cells do not respond appropriately to the trigger (for example a hormonal trigger), just as if PV cells in V1 did not respond appropriately to eye opening. It could be advanced that it is only as the critical period comes to its end that the defects in inscription of certain traces, and thus the absence of structural modification to the neural pathways dependent on the environment, become fixed owing to the closure of the period of plasticity. Work on the cellular mechanisms of critical periods, undertaken by Prochiantz’s laboratory at the Collège de France in collaboration with Takao Hensch at Harvard University, are specifically motivated by the hypothesis that schizophrenia finds its origin in a defect in development:

*Following what is only a hypothesis, this defect would materialize around adolescence during a critical period of the prefrontal cortex’s development […]. [E]ven if the phenotype appears at a late period of development, this does not entail that the “primary causes,” for example one or more epigenetic mutations or modifications, did not take place at an earlier stage, or are hereditary […]. As regards schizophrenia, the hypothesis is that the prefrontal cortex does not adapt to the socialization that follows the hormonal maturation which precedes and accompanies adolescence.* [our translation](Prochiantz, 2014)

This perspective echoes the structural psychoanalytic view according to which, in the decompensation of a psychotic episode, an event precipitates the onset of psychosis, thus making manifest an underlying psychopathological structure that predates the advent of that event. Freud illustrated this idea of a prior structure, with his crystallographic metaphor:

*If we throw a crystal on the floor, it breaks; but not into haphazard pieces, it comes apart along its lines of cleavage into fragments whose boundaries, though they were invisible, were predetermined by the crystal’s structure. Mental patients are split and broken structures of this same kind.* (Freud, 1933)

The psychoanalytic hypothesis of structures thus requires that with psychosis, the psychotic structure, although it is present before the manifest onset of positive symptomatology, will only become apparent ulteriorly following an event that precipitates the onset; just as a crystal that falls and shatters, reveals the lines of its structure in doing so.

## From an excess of plasticity to a disorder in the stability of a sense of self in schizophrenia

In line with an interdisciplinary dialog between psychoanalysis and neuroscience on critical periods and schizophrenia, it seems fruitful to consider the hypothesis of a critical period for the inscription of fundamental traces that have a structuring function in psychic development. Indeed, a disturbance in the usual duration of critical periods for plasticity within the higher cortical pathways, may result in the non-inscription of certain traces linked to interactions with the environment, and thus to the absence of certain representations that could perform an organizing role for the whole of the psychic functioning. If we follow this hypothesis, according to Prochiantz there would be “no difference in nature between the mechanisms that lead to a benign condition like amblyopia – and a devastating illness such as schizophrenia” [our translation] (Prochiantz, 2012). In effect, just as amblyopia is the result of a failure in environmental stimulation within the visual cortex during a critical period for the acquisition of binocular vision (prevented by the reorganization of neuronal networks in that cortex owing to the absence of stimulation), schizophrenia may also be the result of an absence of the inscription of environmental stimuli within the neuronal networks (notably with the prefrontal cortex).

If there is a low PV cells maturation (as in the OTX2-AA mouse), then plasticity opens later and amblyopia can be induced at P100 instead of P30 (Lee et al., 2017).For a psychiatry disease, PV maturation could be too low to permit plasticity and thus adaptation to the post-pubertal adult environment is too difficult. It might also be that the hormonal adult environment is not accompanied by a proper social affective environment (e.g., maternal separation model in mice). The first situation (plasticity is not ready when needed by the environment) is more genetic, and the second one (environment is not appropriate when plasticity is ready) is more environmental. Indeed, any combinations between genetic and environmental factors are possible. So, in schizophrenia, the absence of trace inscription may be linked either to the absence of stimuli, or to the fact that the onset of the critical period had not correctly taken place at the time when the stimuli occurred.

As we have seen, different data support the presence of anomalies in the timing of critical periods in schizophrenia patients. Usually, in the prefrontal cortex, the maturation of the PV cells at adolescence induces a balance between excitation and inhibition circuits. In turn, this terminates the period of plasticity by fixing and rigidifying the neuronal circuits and consolidating E/I balance. In schizophrenia patients however, lacunae in the number of inhibitory synapses, as well as a reduction in the pruning of excitatory pyramidal neuron dendrites, can be observed (Insel, 2010). It is therefore a rise in excitation, to the detriment of inhibition, that takes place within the neuronal circuits of these patients. This in turn prevents the closure of the critical period (or its non-opening or late opening). As we have noted, these data should be considered alongside the immaturity of the PV expressing cells within the prefrontal cortex detected in schizophrenia, since a significant reduction in the expression of parvalbumin within the dorsolateral prefrontal cortex has been observed with this pathology (Chung et al., 2018). A link may therefore exist between this psychiatric illness and anomalies in the mechanisms of onset and closure of critical periods for plasticity. These would be critical periods that were abnormally prolonged, because of a defect of inhibition owing to the belated maturation of the inhibitory circuits (Mauney et al., 2013). Surprisingly then, with schizophrenia patients it would appear that from a physiological perspective it is not a defect, but on the contrary an excess of plasticity – linked to these anomalies in the duration of the critical periods during which cortical plasticity is at its peak – that is observed.

Brain plasticity is certainly an advantage from a biological point of view, since it offers a significant advantage in terms of adaptation. However, stability also plays a major role in the homeostasis of the organism. A tension, inherent to the living organism, seems to emerge between these two poles: the need to adapt to the environment through the neurobiological mechanisms of neuronal plasticity on the one hand; and the necessity for a form of rigidity the makes it possible to ensure the stability over time, of some neuronal pathways. The maturation of the PV cells, that triggers the end of a period of plasticity, thus constitutes an essential stabilizing factor that limits plasticity; a stabilizing factor that, by rigidifying the neuronal circuits, durably fixes certain traces, which then become less subject to remodeling and change. The fact that the critical periods for brain plasticity are framed by precise temporal limits can thus be the precondition for a certain permanence, a stability necessary to the construction of a sense of self that is durable despite the permanent changes to which the organism is prey.

Memory in particular, appears to be one of the factors that enables the introduction of the permanence necessary to the formation of our stability and our sense of identity:

*Indeed, memory requires a minimum of structural permanency, cerebral in particular […]. [D]espite the reality of the morphological and physiological changes, which even in the brains of sapiens continues up to the end, there exists a curb on plasticity that, if left free rein, would erase the history as it was being inscribes in the structure of the brain.* [our translation](Prochiantz, 2012)

Along similar lines, the neuroscientist, Damasio, has also proposed understanding memory as the root of our “autobiographical self.” This is an autobiographical self that relies on our capacity to remember significant events in our personal history, and thus participates in constructing our identity (Damasio, 2000). This understanding can be put in parallel with the words of Prochiantz for whom our capacity to produce narratives around our history – be they close or distant to the reality, “on occasion mythical like the fantasies that surround roots” – are one of the fundamental pillars of the “construction of the individual, of the illusory consciousness that he has of being himself beyond the biological changes, that inscribe themselves, sometimes cruelly, in his flesh, brain included” [our translation] (Prochiantz, 2012). At a cellular level, the critical periods, and notably the maturation of the PV cells that induce their closure, could appear to be one of the neurobiological mechanisms that guarantees a certain fixedness of the neuronal networks, thus ensuring the permanence of those representations or mnesic traces at the root of our identity and our autobiographical selves.

Research by the laboratory of Pico Caroni has demonstrated the importance for long-term memory, as well as for memory consolidation, of a certain population of PV cells within the hippocampus. These neurons, whose early neurogenesis when compared to other PV cells that appear later on in development, are characterized by a strong expression of PV (Donato et al., 2015). The inscription of the traces that determine what Damasio terms autobiographical self (that is, memories of the past, the remembrance of which underpins our sense of identity) therefore involves older PV cells, since these memories necessitate the establishment of a consolidation and a firm stability to become permanently inscribed. Animal models would suggest that in schizophrenia patients a reduced population of these PV cells with a strong expression of PV can be observed – younger cells with a weaker expression of PV being in the majority (Carvalho, 2017). The consequence of this may be disorders in the process of consolidation within the hippocampus, something which has also been observed in other studies of these patients (Genzel et al., 2015).

If we consider the hypothesis of an absence of traces being at the root of psychosis in the light of the neurobiological data on defects in the inhibition of cortical plasticity – defects owing to anomalies in the timeframe of the critical periods in schizophrenia – it is possible that it is particularly the traces and representations at the origin of an autobiographical sense of self that have not been fixed and consolidated in these patients. These traces usually constitute the stable reference point in time that allows for the continuity in our sense of existence. The absence of these traces may thus be at the root of the impairments to the sense of self manifest in the psychopathology of psychosis.

Following the idea of “embodied cognition” developed by Damasio, our feeling of autobiographical self and of self-identity might specifically develop based on certain representations linked to the cartography of our organism, to the mental picture of our own body, within certain areas of the brain. The neuronal mechanisms that generate this representation may appear as the basis for a certain stability of the organism. A stability that Damasio calls “proto-self,” which is at the root of the sense of self. It is from this stable representation of the body, cornerstone of a stable point of reference in time, that the sense of autobiographical self will subsequently develop. This is a sense of autobiographical self from which our sense of identity originates, beyond the permanent changes that model us:

*what might give the brain a natural means to generate the singular and stable reference we call self” […] the possibility that the part of the mind we call self was, biologically speaking, grounded on a collection of non-conscious neural patterns standing for the part of the organism we call the body proper.* (Damasio, 2000)

The stable representation of the body, that comprises “a remarkable degree of structural invariance” (Damasio, 2000), offers a point of reference over long periods of time. Unlike another kind of quasi-instantaneous cartography of the states of the body, which is the object of continuous updating, and on which the mechanisms of homeostatic regulation rely, the stable representation of the body is ensured by other neuronal circuits. These are neuronal circuits with more stable neuronal maps that offer the fixed support of a perennial representation of the general structure of the body. This neuroscientific perspective – in which the neuronal circuits generate a stable representation of the body – represents precisely that reference point – guarantor of the stability and continuity of our conscience and our feeling of existence – that coincides with a psychoanalytic approach.

Following the psychoanalytic understanding, the primordial traces at the basis of the stability of our psychic functioning are the result of the representations and affects that are mobilized during interactions with the parental figures in the Oedipal stage. While, according to the Lacanian theory of the “mirror stage,” it is at an even earlier stage of childhood development that the acquisition of a unified representation of our own body serves as basis for our “self” (Lacan, 2002b). This unified representation of the body does not stem from a representation that is directly linked to the state of the organism, but rather from a stable representation, acquired at an early age, through interactions with the environment and with the adults who themselves mirror back to the child this image of its unity. The image that the child gains of its own body as belonging to a unified whole, is therefore a cornerstone of subjective development, since it is a stable representation that is inscribed as a lasting psychic trace in the subject. It is a representation that will subsequently be the basis for a whole series of other traces or identification, on which the construction of a sense of identity will be formed.

In the Lacanian theory of the mirror stage, it is precisely this representation of a unified body that is absent in psychosis (Lacan, 2002b). This absence of a fundamental trace, which is the source of stability for the development of subjectivity and awareness of self, is made apparent with the onset of psychosis by the appearance of symptoms connected with an altered perception of the body. The experience of fragmentation, the strong presence of hypochondriac thematics, and coenesthetic hallucinations that are characteristic of the clinical picture of schizophrenia, may be understood as a consequence of this absence of a unifying and organizing representation (Lacan, 1997).

Freud had brought to light the existence of this altered perception in the representation of the body with his study of the Schreber case. In the initial phases the clinical picture was dominated by coenesthetic hallucinations and ideas of a hypochondriac nature (such as the feeling of no longer having a stomach, or of having certain organs such as the lungs damaged or even destroyed; Freud, 1911c). The introduction of narcissism would then enable Freud to disentangle the two phases of the psychotic process that consisted of a twofold libidinal movement. The first phase of psychosis consisted in a massive withdrawal of libidinal cathexis, and of all the psychic energy, from external objects. The libido flows back exclusively on the ego and the subject’s own body. This first phase was characterized by the fact that the patient (notably those with schizophrenia) “…withdraws his interest from the external world completely…” (Freud, 1911c). The negative symptomatology of schizophrenia, such as being withdrawn, blunted affect, apragmatism, and deteriorations in representations of the body (coenesthetic hallucinations, hypochondria, and dysmorphophobia), are characteristic of this initial phase.

Freud postulated that this first phase is common to all forms of psychosis. However, in order to fight against this movement of libidinal withdrawal, some patients put in place a second process that would consist in an attempt to reinvest external objects with the libido. This second phase finds its expression in productive symptomatology such as auditory hallucinations and delusional ideas. In this perspective, Freud describes delusion as an “attempt at recovery” process (Freud, 1914c).

In the interdisciplinary model of schizophrenia as an “absence of trace,” the delusional ideas can be equated to an attempt by the subject to produce new traces, new representations (R) by way of the delusion. This is in order to pacify the altered experience of the somatic states and the fear of fragmentation. It is an attempt, in the aftermath, to make up for the traces that had not been inscribed during development.

Psyochanalysis and neuroscience come together around the shared observation that certain traces and representations, lastingly and durably inscribed, constitute a reference point for our development and ensure a stability on which the sense of self, identity, and autobiographical consciousness can be built despite the continuous changes to which the organism is subject. The existence of critical periods of cerebral plasticity that are strictly delimited in time, constitutes an indispensable condition for the acquisition of a sense of self.

The anomalies observed in the molecular and cellular mechanisms that regulate the timeframes of the critical periods seen in schizophrenia patients, can therefore, owing to an excess of plasticity, have as consequence an absence of inscription or of consolidation of those traces that result from interactions with the environment. These are traces that offer a reference point, in terms of stability, for psychic development and the acquiring of a sense of autobiographical self. As we saw, this was the case also for the acquisition of a stable representation of a picture of the body, and according to Freudian theory for the psychic traces resulting from the resolving of the Oedipus complex. The absence of these traces, that usually form a reference point for everything that concerns procreation, access to parentality and maternity, and sexual identity in adult life, may in this way lead to the symptomatology that is characteristic of the clinical picture for schizophrenia; in particular symptoms connected with disorders in the perception and representation of the body, as well as an impairment of sense and awareness of self.

## The reopening and reclosing of critical periods in schizophrenia: A therapeutic horizon

The dialog between psychoanalysis and neuroscience around the hypothesis of an absence of trace and anomalies in critical periods in schizophrenia may also open new horizons for treatment. Based on research into the importance of critical periods in the process of neurodevelopment at work in schizophrenia, a better understanding of the molecular mechanisms connected with the maturation of PV cells that conditions the onset and closure of critical periods, points to significant new therapeutic possibilities. Recent research on the molecular mechanisms of critical periods have brought to light the possibility of acting upon these mechanisms through pharmacological treatments, perhaps even reopening and reclosing certain periods of plasticity in adults. This research has raised the hope of being able to repeat certain stages of brain development, through the reopening and then closing of windows of plasticity. In particular, experiments have succeeded in reversing experimentally induced amblyopia in mice, enabling individual with amblyopia to regain normal binocular vision after the closure of the critical period for acquiring binocular vision. This was done by reopening a window of plasticity through the administering of a pharmacological drug within the visual cortex of the mice.

Several neuroscientific experiments have proposed different strategies to enable the manipulation and reopening of periods for developmental plasticity (Bavelier et al., 2010). The preferred approaches focus generally on neuromodulators such as dopamine, norepinephrine, or acetylcholine, as there are advantages to using drugs that are already approved for distribution (Bavelier et al., 2010). Experiments undertaken in the laboratory of Hensch have identified, in mice, the role played by a protein called Lynx1, which contributes to the plasticity inhibiting mechanism, and induces the closure of critical periods. The expression of Lynx1 contributes to maintaining the stability of mature cortical networks, as well as the suppression of molecular breaks that allow a modulation of the balance between the excitatory and inhibitory circuits that reactivate visual plasticity. For this reason, it represents an important avenue for the treatment of amblyopia, and opens up further interesting therapeutic prospects (Morishita et al., 2010).

Other similar studies have focused on the role of serotonin and norepinephrine in adult plasticity. These studies have successfully induced the reopening of critical periods by modulating the concentration of these neurotransmitters within the cortex (Spolidoro et al., 2009). Other scientists have for their part focused on another route towards the reopening of critical periods, notably through research into the role played by PNNs in the regulation of cortical periods of plasticity. Based on a study of the makeup of these extracellular matrices, which are organized in networks around neurons, Tommaso Pizzorusso’s team have shown that their deterioration induced by hydrolysis with chondroitinase-ABC (chABC) leads to a reactivation of cortical plasticity in rats, thus enabling the reopening of the critical period for binocular vision (Pizzorusso et al., 2002; Harauzov et al., 2010).

The research done on the homeoprotein OTX2 that is internalized by the PV expressing basket neurons, by Prochiantz’s team in collaboration with Hensch’s laboratory, offers another interpretation of these results. The internalization of OTX2 is necessary and sufficient for opening then closing the critical period of plasticity in the visual cortex of mice. The teams have demonstrated that it is the perineuronal networks that surround the PV cells which capture the OTX2, by way of binding sites specific to the GAG present in that homeoprotein (Beurdeley et al., 2012). By identifying the molecules involved in these binding sites, the scientists have managed to demonstrate that the hydrolysis of the perineuronal networks by the chABC reduces the quantity of OTX2 present in the PV cells. This study has also shown that the direct infusion of a peptide, RK-pep, disturbed in a similar way the binding of OTX2 to the PV cells, reducing the expression of PV as well as the presence of PNN, thus reopening plasticity in the visual cortex of mice (Beurdeley et al., 2012). In the adult animals with amblyopia this momentary reopening of plasticity allowed a recovery of binocular vision.

There exists, therefore, different ways of reopening the critical periods for plasticity, using pharmacological means to act upon quantities of OTX2 (Despras et al., 2013; Testa et al., 2019). Modifying the binding of the homeoprotein OTX2 to the PNNs through pharmacological means, constitutes a potential new therapeutic tool. Such a tool might make it possible to restore the cortical plasticity not only in the visual cortex of humans, but also in other regions of the cerebral cortex. Based on the results obtained with amblyopia, scientists to come will be able to envisage new therapeutic horizons for other neurodevelopmental pathologies, and in particular for psychiatric pathologies such as schizophrenia.

Some studies have already sought to apply these ideas to animal models of schizophrenia, and have obtained significant results. Based on the hypothesis that the critical periods within different cortical regions are regulated by common mechanisms involving the maturation of PV basket cells, scientists have attempted to induce experimentally a structural, functional, or molecular lifting of the breaks that prevent plasticity in the areas implicated in the physiopathology of schizophrenia. In particular, Felipe Gomes and his team have looked at the critical period for the development of the ventral hippocampus that takes place in adolescence. This constitutes a period that is particularly sensitive to exposure to environmental stress factors. These can ultimately lead to a reduction in the physiological regulation of stress, and become a risk factor for schizophrenia (Gomes et al., 2019). The same team has also used sodium valproate, which has a strong inhibiting action, and which has been recognized for inducing a reopening of critical periods. Results have shown that administering valproate in adult mice enables a reopening of the critical period for vulnerability to stress, inducing a state of physiological maturation similar to that of adolescent mice. These data evidence the fact that adolescence is, from a physiological point of view, a critical period for acquiring a vulnerability to stress; a period that can be restored in adults through pharmacological means. These results are particularly important for our understanding of the physiopathology of schizophrenia, where this critical period could constitute a period of particular sensitivity to the impact of environmental factors that can contribute to the expression of a schizophrenia phenotype.

In so far as critical periods can be pharmacologically modulated, the experimental lifting of the various breaks on brain plasticity can be seen as a significant therapeutic step forward for schizophrenia symptoms. In the wake of the interdisciplinary hypothesis of an absence of trace at the root of this pathology, it is possible to envisage that a reopening of the critical periods for plasticity in certain regions of the brain could enable the replaying of some developmental processes; processes that had been disrupted or hindered the first time around. Thus, ultimately, it may be possible to achieve a re-inscription or a consolidation of certain traces that had not been stabilized during development.

If we follow the hypothesis that the lack of inhibition linked to the abnormalities in the maturation of the PV neurons that regulate critical periods could therefore, in the case of schizophrenia, be associated with an excess of plasticity, a therapeutic strategy could be the closure of the plasticity, with the aim of consolidating the traces that could not be stabilized during development.

But if we concentrate on a defective maturation of PV cells, following the hypothesis that maturation could be so low that the first threshold (plasticity opening) is not even passed (which means that the schizophrenia phenotype would be a consequence of an absence of plasticity when needed to repose to an environmental change), the therapeutic strategy will be different. The objective will then be to open critical period of plasticity, provide appropriate environment stimulation, and then close the critical period. This process would allow a long-lasting traces inscription.

If we consider schizophrenia as a neurodevelopmental pathology, linked in particular to anomalies in the duration of critical periods (anomalies that prevents the consolidation and the stabilization of certain representations which have a regulating and homeostatic function in psychic development) we could suppose that the reintroduction of plasticity within certain regions of the adult brain could allow, when associated with certain environmental stimuli such as psychotherapy, for a consolidation of the association between certain traces with a homeostatic function, thus making them more stable and perennial. This could be a way of attempting to replay the fundamental *R-S* tie that had not been the object of a lasting inscription during the subject’s development. The potential for a psychological treatment would thus be increased, insofar as the return to a certain developmental plasticity would make it possible to inscribe and anchor in a more durable way the representations or signifiers evoked by the patient during sessions. Neurobiology’s search for a chemical means of reopening critical periods would thus constitute a new therapeutic perspective for psychosis. It may even be an important trump card that would increase the effects of a talking cure, becoming a significant pharmacological aid in the psychoanalytic treatment of psychosis.

## Conclusion

The interdisciplinary hypothesis of an absence of trace in psychosis, guided by research on anomalies in critical periods observed with schizophrenia, makes it possible to reconsider this pathology through an analogy with a developmental pathology such as amblyopia. Thus, seeing schizophrenia as consequent to the absence of inscription of certain fundamental traces linked to early interactions with the environment. Even when these early interactions, and the social and affective stimuli they imply, have taken place, an anomaly in the timeframe of the critical periods can prevent the inscription and the fixing of the neuronal traces stemming from these interactions. Cerebral anomalies identified by brain imaging in schizophrenia patients, may therefore be (as is the case with amblyopia) the result of this absence of reorganization in the neuronal networks that takes place through the influence of experience and environmental stimuli.

Research into the molecular mechanism that regulate the onset and closure of critical periods for brain plasticity have brought to light the regulation of these mechanisms – for example through the expression of the transcription factor OTX2, and by the PNN that surround the PV neurons. This demonstrates that genetic factors, as well as environmental ones, can act upon the timing of critical periods. The dialog between psychoanalysis and neuroscience that has developed around the cellular anomalies observed in the regulation of critical periods in schizophrenia patients, fits into a perspective that goes beyond the narrow opposition between genetic and environmental factors that has been linked to the etiology of this pathology.

Finally, neurobiological research into the pharmacological reopening of critical periods for plasticity represent a promising new therapeutic horizon, in that it may open the way to a reintroduction of developmental plasticity within certain cortical regions, in adult schizophrenia patients. Combined with environmental stimuli, this reopening could make it possible to consolidate or reinscribe certain associations of traces that had initially not been inscribed in a stable and perennial way during the subject’s development. If these possibilities remain to date only in the realms of fiction, they could, ultimately, lead to new interdisciplinary therapeutic strategies between psychoanalysis and neurobiology, in the treatment of schizophrenia. The possibilities for a psychological treatment, could be vastly increased using chemical methods aimed at reintroducing a degree of developmental plasticity. The dialog between psychoanalysis and neuroscience centered on the role of critical periods in schizophrenia, shows itself to be rich in promise as regards new approaches in the treatment of this pathology.

## Data availability statement

The original contributions presented in the study are included in the article/supplementary material, further inquiries can be directed to the corresponding author.

## Author contributions

JT is the main contributor of this manuscript as part of her postdoctoral research. FA and PM supervised the research, contributed to the conception and development of the research, and revised critically the manuscript for intellectual content. All authors contributed to the article and approved the submitted version.

## Conflict of interest

The authors declare that the research was conducted in the absence of any commercial or financial relationships that could be construed as a potential conflict of interest.

## Publisher’s note

All claims expressed in this article are solely those of the authors and do not necessarily represent those of their affiliated organizations, or those of the publisher, the editors and the reviewers. Any product that may be evaluated in this article, or claim that may be made by its manufacturer, is not guaranteed or endorsed by the publisher.
